# The metal hyperaccumulators from New Caledonia can broaden our understanding of nickel accumulation in plants

**DOI:** 10.3389/fpls.2013.00279

**Published:** 2013-07-26

**Authors:** Tanguy Jaffré, Yohan Pillon, Sébastien Thomine, Sylvain Merlot

**Affiliations:** ^1^Laboratoire de Botanique et D'écologie Végétale Appliquées, Herbarium NOU, UMR AMAP, Institut de Recherche pour le DéveloppementNouméa, New Caledonia; ^2^Tropical Conservation Biology and Environmental Science, University of Hawai'i at HiloHilo, HI, USA; ^3^Saclay Plant Sciences Labex, Institut des Sciences du Végétal, Centre National de la Recherche ScientifiqueGif-sur-Yvette, France

**Keywords:** nickel hyperaccumulator, New Caledonia, ultramafic soils, adaptation, comparative transcriptomic

## Abstract

While an excess of metals such as zinc, cadmium or nickel (Ni) is toxic for most plants, about 500 plant species called hyperaccumulators are able to accumulate high amounts of these metals. These plants and the underlying mechanisms are receiving an increasing interest because of their potential use in sustainable biotechnologies such as biofortification, phytoremediation, and phytomining. Among hyperaccumulators, about 400 species scattered in 40 families accumulate Ni. Despite this wide diversity, our current knowledge of the mechanisms involved in Ni accumulation is still limited and mostly restricted to temperate herbaceous Brassicaceae. New Caledonia is an archipelago of the tropical southwest pacific with a third of its surface (5500 km^2^) covered by Ni-rich soils originating from ultramafic rocks. The rich New Caledonia flora contains 2145 species adapted to these soils, among which 65 are Ni hyperaccumulators, including lianas, shrubs or trees, mostly belonging to the orders Celastrales, Oxalidales, Malpighiales, and Gentianales. We present here our current knowledge on Ni hyperaccumulators from New Caledonia and the latest molecular studies developed to better understand the mechanisms of Ni accumulation in these plants.

## Introduction

New Caledonia is an archipelago located in the southwest Pacific off the east coast of Australia (20–23 S, 164–167 E). New Caledonia separated from Gondwana in the Cretaceous, and after a submersion during Paleocene and Eocene, the main island emerged 37 million years ago (Pelletier, 2006) and was likely exclusively re-colonized by plants through long-distance dispersal (Grandcolas et al., 2008; Cruaud et al., 2012; Pillon, 2012). At the time of its emersion it was probably entirely covered with ultramafic rocks that still occupy 34% (5500 km^2^) of the surface of the main island. The weathering of these rocks produced a variety of soils that have a very low level of phosphorus, potassium, calcium and high level of magnesium and heavy metals including nickel (Ni), cobalt and chromium (Jaffré, 1980; Brooks, 1987). New Caledonia harbors a rich and original vegetation likely resulting from the adaptation of plants over a long period of time to these challenging edaphic conditions. New Caledonia is renowned for its rich, endemic and threatened vascular flora (Morat et al., 2012) and is therefore considered as one of the world biodiversity hotspots (Myers et al., 2000). In particular, 2145 species, among which 80% are endemic, are developing on these metal rich soils (Jaffré et al., 2009). As a consequence, New Caledonia has also been listed among the main hotspots for metallophytes (Whiting et al., 2004).

## Metal hyperaccumulators from new Caledonia: history and current knowledge

Unusually high concentration of nickel (Ni) in plants were first reported in the Italian Brassicaceae *Alyssum bertolonii* (Minguzzi and Vegnano, 1948) and later in *Hybanthus floribundus* (Violaceae) from Australia (Severne and Brooks, 1972). Ni concentration exceeding 1% (dry weight) in New Caledonian plants growing on ultramafic soils was first reported in *Psychotria gabriellae* (previously known as *P. douarrei*, Rubiaceae), *Hybanthus austrocaledonicus*, *H. caledonicus* (Violaceae), *Geissois pruinosa* (Cunoniaceae), and *Homalium guillainii* (Salicaceae) (Brooks et al., 1974; Jaffré and Schmid, 1974). These plants accumulating more than 1% Ni were then qualified as “hypernickelophore” (Jaffré and Schmid, 1974). The more widely used term Ni “hyperaccumulator” was first coined by Jaffré et al. (1976) for the description of *Pycnandra acuminata* (previously known as *Sebertia acuminata*, Sapotaceae) in which Ni represents up to 25% of the latex dry weight. The term ‘hyperaccumulator’ was later defined more precisely by Brooks et al. (1977) to qualify plants with a concentration of Ni above 0.1% (1000 ppm) in dried leaves and extended to other metal accumulators, although with different thresholds (Baker and Brooks, 1989; van der Ent et al., 2013a). Following these pioneering studies, a large number of species that hyperaccumulate Ni were described from the New Caledonian flora (Jaffré, 1977a, 1980; Jaffré et al., 1979a,b; Kersten et al., 1979; Amir et al., 2007). Today, we consider that 65 Ni hyperaccumulator taxa scattered in 12 plant families have been identified (see Supplementary Table), placing New Caledonia, along with Cuba that contains about 140 hyperaccumulators (Reeves et al., 1996, 1999), as one of the most important reservoirs of Ni hyperaccumulators. We can predict that the number of Ni hyperaccumulators in New Caledonia will slightly increase in the coming years considering that the hyperaccumulator status of known taxa (e.g., *Pancheria ferruginea*, Cunoniaceae; *Capparis artensis*, Capparaceae) was revealed by recent analyses and that new hyperaccumulator taxa, such as *Pycnandra caeruleilatex* (Swenson and Munzinger, 2010), are still being described. The New Caledonian flora also contains manganese hyperaccumulators (above 1% of dry weight) such as *Denhamia fournieri* (previously known as *Maytenus fournieri*, Celastraceae), *Alyxia poyaensis* (previously a subspecies of *Alyxia rubricaulis*, Apocynaceae), *Virotia neurophylla* (previously known as *Macadamia neurophylla*, Proteaceae), and *Garcinia amplexicaulis* from the Clusiaceae family (Jaffré, 1977b, 1979; Fernando et al., 2008, 2010). Interestingly, *Denhamia fournieri* is also able to hyperaccumulate Ni when growing on ultramafic soil.

## Taxonomic distribution of nickel hyperaccumulators and evolution of the hyperaccumulator trait

**Figure 1:**
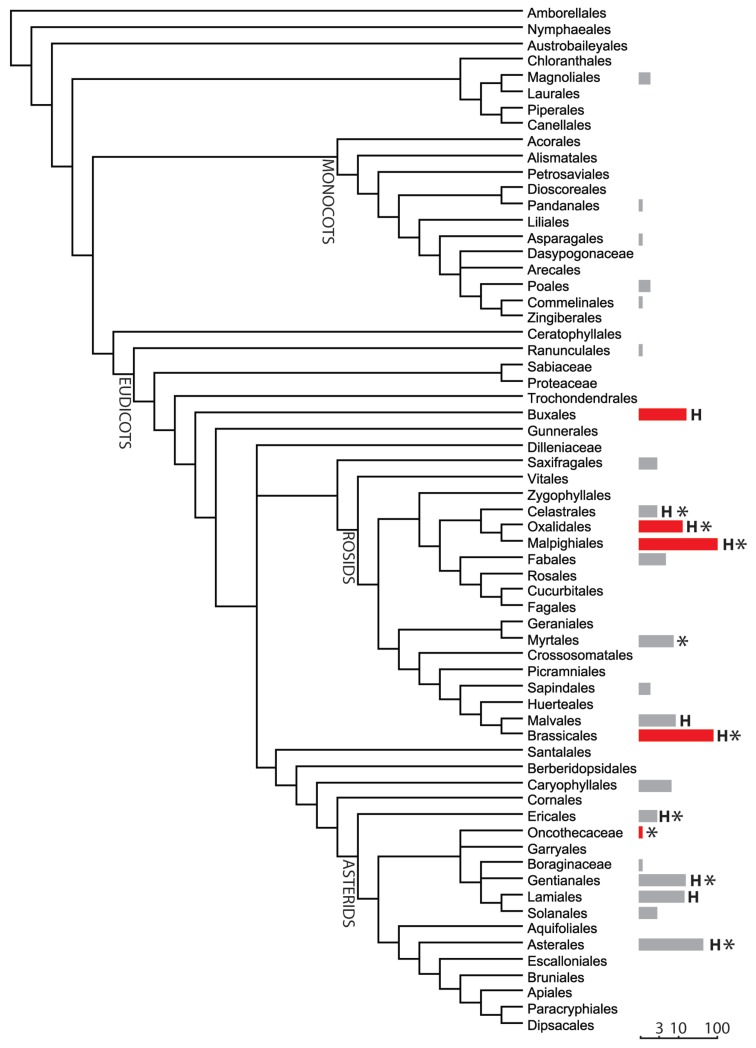
**Phylogenetic distribution of nickel hyperaccumulators using APG III classification.** The number of Ni hyperaccumulator in each order is presented as a bar (logarithmic scale). The red bar indicates that the observed number of hyperaccumulators significantly exceed the theoretical number (hyperaccumulating species randomly distributed using a binomial law with a Bonferroni correction for multiple tests). H, indicates the presence of hypernickelophore species accumulating more than 10,000 ppm nickel. ^*^, indicates the presence of hyperaccumulators from New Caledonia.

Worldwide about 400 nickel (Ni) hyperaccumulators scattered in more than 40 families have been reported almost exclusively in angiosperms (Verbruggen et al., 2009; Krämer, 2010), however at least one fern of the *Adiantum* genus (Brooks et al., 1990), and several epiphytic bryophytes and liverworts growing on hyperaccumulating shrubs in New Caledonia can be considered as Ni hyperaccumulators (Boyd et al., 2009), suggesting that Ni hyperaccumulation could have appeared early in the evolution of terrestrial plants. Within angiosperms, Ni hyperaccumulators are mainly found in Eudicots, although few examples in Magnolianae and Monocots have been described (Figure 1). Ni hyperaccumulators are taxonomically scattered, nevertheless phylogenetic trends can be observed (Pillon et al., 2010). A third of the Ni hyperaccumulators belongs to the COM clade (Celastrales, Oxalidales, and Malpighiales) which form part of Rosids that account for two third of the Ni hyperaccumulating species. Although this character appeared many times in the course of evolution, it tends to come out more often in certain lineages. In New Caledonia, 83% of the Ni hyperaccumulators are from the COM clade while only one hyperaccumulator from each of the orders Brassicales and Asterales have been identified (see Supplementary Table). The explanation for this bias remains not fully understood.

There are still only few phylogenetic studies that have investigated how nickel hyperaccumulating species evolved from non-accumulating ancestors, and whether the ability to grow on ultramafic soils is a prerequisite for this character to appear. In some genera, such as Australian *Stackhousia* (Burge and Barker, 2010) or Californian *Streptanthus* (Mayer and Soltis, 1994), Ni hyperaccumulation is a rare character found only in one species, although several other related species are growing on Ni-rich soils. This is also the case in New Caledonian *Psychotria*, in which *P. gabriellae* is the only Ni hyperaccumulator while 60 *Psychotria* species, including *P. semperflorens*, are present on ultramafic soils (Jaffré, 1980; Baker et al., 1985). In contrast, in New Caledonian *Phyllanthus* (Euphorbiaceae; Kersten et al., 1979), and in Cuban *Buxus* (Buxaceae; Reeves et al., 1996), a significant numbers of species growing on ultramafic soils hyperaccumulates Ni. In Meditterranean *Alyssum* sect. *Odontarrhena* (Cecchi et al., 2010) and in New Caledonian *Geissois* (Cunoniaceae; Jaffré et al., 1979a), almost all species occurring on ultramafic soils hyperaccumulate nickel, but they are phylogenetically entangled with non-accumulating species restricted to non-ultramafic soils (Cecchi et al., 2010; Pillon et al., unpublished). In the Carribean genus *Leucocroton* (Euphorbiaceae), mostly diversified on Cuba, all 26 species are restricted to ultramafic soils and hyperaccumulate nickel (Jestrow et al., 2012). Therefore, the link between the adaptation to ultramafic soils and Ni hyperaccumulation shows some striking variations that are also found within New Caledonian hyperaccumulators. Both characters seem to appear recurrently in some genera in distant areas, e.g., in *Psychotria* and *Phyllanthus* from New Caledonia, Cuba and other regions (Reeves, 2003). Although no specific study have addressed this question, molecular phylogenetic analyses indicated that *Phyllanthus* and *Psychotria* species, including nickel hyperaccumulators, from New Caledonia and the Caribbean region belong to distant clades within these genera (Andersson, 2002; Kathriarachchi et al., 2006; Barrabé, 2013).

Parallel evolution of the characters at broad taxonomical and geographical scales may be facilitated by an ancestral fortuitous pre-adaptation, that can be called exaptation, to ultramafic soils (Pillon et al., 2010). At a smaller scale, it may have evolved only once and then have been transmitted horizontally through hybridization and introgression between closely related species, as observed for the adaptation to ultramafic soils (Pillon et al., 2009a,b), in a process that could be qualified as collective evolution (Morjan and Rieseberg, 2004). The great diversity of plants adapted to ultramafic soils and that can hyperaccumulate Ni in New Caledonia, combined with its geographic isolation, makes this archipelago an ideal system to study the evolution of these two characters.

## Molecular studies on new Caledonian nickel hyperaccumulators

Our current understanding of the hyperaccumulation of metals in plants has been recently recapitulated in excellent review articles (Verbruggen et al., 2009; Krämer, 2010; Rascio and Navari-Izzo, 2011). It now clearly appears that molecular mechanisms involved in metal chelation and transport play key roles in this remarkable adaptation. However, only few mechanisms involved in nickel (Ni) hyperaccumulation have been identified at the molecular level, and this knowledge only originates from studies of hyperaccumulators from the Brassicaceae family. The study of Ni hyperaccumulators from other plant families would therefore likely bring new and original information on the mechanisms involved in Ni hyperaccumulation in plants.

After the identification of Ni hyperaccumulators in New Caledonia in the early 70's, one of the first questions was to identify molecules that are able to chelate Ni and therefore play a role in detoxification. Early analyses of New Caledonian hyperaccumulators revealed that Ni was essentially associated with organic acids. Citrate was identified as a main ligand for Ni in the latex of *Pycnandra acuminata* and in leaves of several hyperaccumulators in the *Homalium* and *Hybanthus* genera (Lee et al., 1977, 1978), while 63% of Ni was bound to malate in leaves of *Psychotria gabriellae* (Kersten et al., 1980). The existence of Ni-citrate complexes in several hyperaccumulators was largely confirmed since then in several studies that use state-of-the art techniques of chromatography and mass spectrometry (Sagner et al., 1998; Schaumlöffel et al., 2003; Callahan et al., 2008, 2012). These recent studies also revealed the presence of Ni-nicotianamine (NA) complexes in most of the New Caledonian species that were studied, including *P. acuminata* and *P. gabriellae* (Schaumlöffel et al., 2003; Callahan et al., 2012). Callahan et al. (2008, 2012) developed a metabolic profiling approach based on GC-MS to identify putative ligands whose abundance in New Caledonian hyperaccumulators correlates with Ni concentration. Putative Ni-metabolite complexes were then directly identified using LC-MS. These analyses identified 2,4,5-trihydroxy-3-methoxy-1,6-hexan-dioic acid as a new ligand for Ni in the latex of *P. acuminata*. In contrast to *Alyssum* and *Noccaea* hyperaccumulators (Krämer et al., 1996; Persans et al., 1999), no correlation between the amino acid histidine and Ni was observed so far in the New Caledonian hyperaccumulators. Rather, a significant correlation was observed with aspartic acid, which has a relatively high association constant for Ni (lg *K* = 7.2), but LC-MS analyses failed to directly detect Ni-aspartic acid complexes in these species. Analyses of *P. gabriellae* samples revealed several Ni-organic acid complexes, including malonic and maleic acids, but did not confirm the existence of a complex with malic acid. In addition, the authors pointed out that several intense Ni-containing ions could not be identified by LC-MS analysis from *P. gabriellae* samples (Callahan et al., 2012). These studies confirmed that Ni forms complexes with a plethora of organic acids, which could be of biological significance for the storage of Ni in vacuoles. NA that is able to bind Ni with a high affinity (lg *K* = 16), seems to be an ubiquitous Ni ligand in dicotyledon hyperaccumulators and may play an important role in cytoplasmic Ni detoxification and long range transport (Kim et al., 2005; Pianelli et al., 2005; Gendre et al., 2007). In contrast, histidine might not play a significant role in Ni homeostasis in New Caledonian hyperaccumulators and the presence of Ni-complexes with other amino acids still needs to be confirmed. Additional metabolomic studies using alternative extraction and separation methods will be required to validate and identify new Ni-complexes from New Caledonian hyperaccumulators. Ultimately, the speciation of Ni in New Caledonian hyperaccumulators should be also confirmed *in vivo*, for example using X-ray spectrometric methods (Montarges-Pelletier et al., 2008).

**Figure 2:**
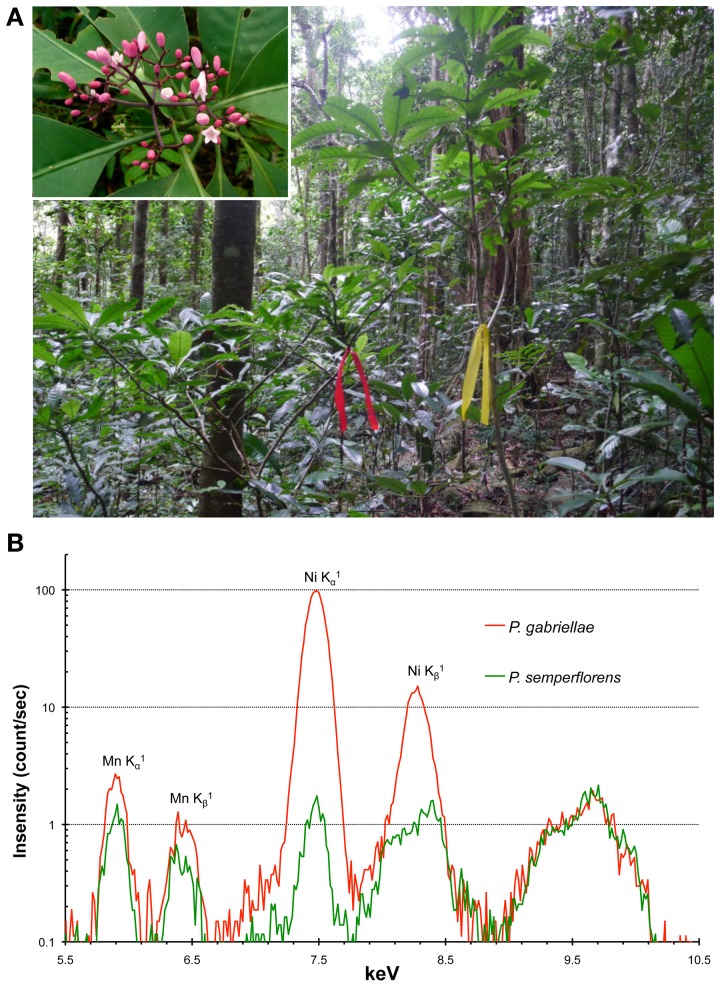
***Psychotria gabriellae* and *Psychotria semperflorens* as a model system to study nickel hyperaccumulation.** The Ni hyperaccumulator *P. gabriellae* (red tag) and the closely related non-accumulator *P. semperflorens* (yellow tag) are living in sympatry in rain forest on ultramafic soil in New Caledonia **(A)**. Both species are 1–3 meters shrub with very similar pink flowers (top right insert: *P. semperflorens* inflorescence). In natural growth condition, *P. gabriellae* accumulates up to 4% Ni in leaves while *P. semperflorens* accumulates approximately 100 times less Ni. These differences can be semi-quantitatively visualized on XRF spectra **(B)**.

In parallel to these metabolomic studies, it is also important to identify genes coding for key enzymes in the biosynthesis of Ni ligands and for Ni membrane transporters. However, no nucleotide sequences were available until recently for any of the New Caledonian hyperaccumulators. The recent development of Next Generation Sequencing (NGS) technologies and the creation of a molecular biology platform in New Caledonia (Majorel-Loulergue et al., 2009), allowed us to initiate the sequencing of the *P. gabriellae* leaf transcriptome using Roche GS-FLX titanium technology. We obtained more than 500,000 reads that are publicly available at the European Nucleotide Archive under accession number ERP001334. The *de novo* assembly of the sequence reads generated approximately 30,000 contigs that represented the first EST data set for *P. gabriellae*. We are currently using these original sequences to identify putative Ni transporters from the NRAMP, IREG/FPN, YSL, ZIP/IRT families (Verbruggen et al., 2009; Krämer, 2010; Rascio and Navari-Izzo, 2011), that might be involved in nickel tolerance and hyperaccumulation in leaves (Merlot et al., submitted). To identify additional genes important for Ni accumulation, it will be necessary to sequence the root transcriptome of *P. gabriellae*. In parallel, the seed transcriptome of *P. gabriellae* was sequenced by the 1KP project in collaboration with the University of New Caledonia (www.onekp.com/index.html). Also, because it is not presently possible to genetically manipulate this species, one strategy to target genes of interest is to compare its transcriptome with that of a phylogenetically related, non-accumulating species and thereby identify genes whose expression is linked to the hyperaccumulation trait. *Psychotria semperflorens*, which lives in sympatry with *P. gabriellae* in rain forest on ultramafic soil but does not accumulate Ni (Figure 2), represents an excellent candidate to conduct comparative transcriptomic studies. Interestingly, the flora of Cuba contains at least five Ni hyperaccumulators of the *Psychotria* genus including *P. costivenia* and *P. clementis* (Reeves et al., 1999). The comparison of the molecular mechanisms involved in Ni adaptation and hyperaccumulation in *Psychotria* species from the flora of Cuba and New Caledonia that have different origins, will help to understand the evolution of these traits and possibly reveal the genetic basis of the exaptation to ultramafic soils. A similar approach using New Caledonian species could be used to study the evolution of Ni hyperaccumulation mechanisms in the Phyllanthaceae genus *Phyllanthus* (including *Glochidion*) that is also present in Cuba and Indonesia (Kersten et al., 1979).

## Concluding remarks

The hyperaccumulation of nickel (Ni) is a complex trait that appeared several times during evolution of plants. New Caledonia hosts a rich and phylogenetically diverse set of Ni hyperaccumulators and offers significant opportunities to broaden our knowledge of the molecular mechanisms involved in Ni hyperaccumulation that was mostly studied so far in European Brassicaceae. Today, such studies are realistic considering the rapid development of NGS technologies that give access to virtually all plant genomes and also because of the relatively good knowledge of the diversity of New Caledonian hyperaccumulators. The identification of genes involved in Ni accumulation in these species will be important to identify conserved and divergent mechanisms involved in Ni hyperaccumulation and also adaptation to ultramafic soils. We think these results will support the development of phytoremediation and phytomining technologies that are particularly important in regions such as New Caledonia (Losfeld et al., 2012) and Indonesia (van der Ent et al., 2013b), where the nickel mining industry and the economical development are often opposed to the protection of environment and endangered flora. In every respect, future studies on New Caledonian hypernickelophore accumulating more than 1% Ni and producing a high biomass such as species of *Geissois* (Fogliani et al., 2009) or *Homalium*, being related to *Populus*, will be of particular interest (Chaney et al., 2007).

### Conflict of interest statement

The authors declare that the research was conducted in the absence of any commercial or financial relationships that could be construed as a potential conflict of interest.
