# Supplementary material for: The metal hyperaccumulators from New Caledonia can broaden our understanding of nickel accumulation in plants
Source: Front Plant Sci. 2013 Jul 26;4:279. doi: 10.3389/fpls.2013.00279 (PMC3724167; doi:10.3389/fpls.2013.00279)
Supplement: Supplementary file 1 [file DataSheet1.DOCX]

**Supplementary Table: Nickel hyperaccumulator taxa from New Caledonia**

| **Taxa** | **Number of nickel (Ni) analyses** | | | **[Ni] range** (ppm) | **Ecology**^a^ | **References** |
| --- | --- | --- | --- | --- | --- | --- |
|  | Total | [Ni] ppm ≥1,000 & < 10,000 | [Ni] ppm ≥ 10,000 |  |  |  |
| **CELASTRALES** |  |  |  |  |  |  |
| Celastraceae |  |  |  |  |  |  |
| *Denhamia fournieri var fournieri (Maytenus fournieri)* | 36 | 2 | 0 | 68 - 1,575 | M | new data |
| *Peripterygia marginata* | 44 | 2 | 0 | 23 - 1,800 | U | Jaffré, 1980; new data |
| **Malpighiales** |  |  |  |  |  |  |
| EUPHORBIACEAE |  |  |  |  |  |  |
| *Baloghia drimifolia (Baloghia sp)* | 7 | 6 | 0 | 700 - 5,375 | U | Jaffré, 1980 |
| *Cleidion vieillardii var vieillardii (C. lasiophyllum)* | 12 | 6 | 0 | 134 - 4,250 | M | Jaffré, 1980 |
| PHYLLANTACEAE |  |  |  |  |  |  |
| *Phyllanthus aeneus var aeneus* | 27 | 18 | 0 | 9 - 2,498 | U | Kersten *et al*., 1979; new data |
| *Phyllanthus barouaensis* | 4 | 1 | 3 | 4,320 - 15,000 | U | new data |
| *Phyllanthus bupleuroides var ngoyensis (P. ngoyensis)* | 3 | 3 | 0 | 8,240 - 9,950 | U | Kersten *et al*., 1979 |
| *Phyllanthus bupleuroides var latiaxalis* | 1 | 1 | 0 | 1,920 | U | new data |
| *Phyllanthus chamaecerasus var vieillardii (P. cataractarum)* | 14 | n.a.^b^ | 0 | 2 - 1,450 | M | Kersten *et al*., 1979 |
| *Phyllanthus chrysanthus (all varieties)* | 3 | 3 | 0 | 240 - 1,180 | M | Kersten *et al*., 1979 |
| *Phyllanthus favieri var favieri (serpentinus pro-parte)* | 10 | 0 | 10 | 10,003 - 42,213 | U | Jaffré, 1977; Kersten *et al*., 1979; Amir *et al.*, 2007 |
| *Phyllanthus favieri var kaalaensis* | 1^c^ | - | - | ≥ 1000 ^c^ | U | new data |
| *Phyllanthus kanalensis* | 6 | 6 | 0 | 26 - 1,090 | M | Kersten *et al*., 1979 |
| *Phyllanthus luciliae (serpentinus pro-parte)* | 2 | 0 | 2 | 10,000 - 33,916 | U | new data |
| *Phyllanthus memaoyaensis* | 2 | 0 | 2 | 24,980 - 29,272 | U | new data |
| *Phyllanthus montouzieri* | 7 | 2 | 0 | 570 - 1100 | U | new data |
| *Phyllanthus parangoyensis (serpentinus pro-parte)* | 2 | 0 | 2 | 10,000 - 26,823 | U | Kersten *et al*., 1979; new data |
| *Phyllanthus peltatus* | 9 | 6 | 0 | 161 - 2,830 | U | Kersten *et al*., 1979; new data |
| *Phyllanthus serpentinus (P. Serpentinus pro-parte)* | 7 | 1 | 6 | 3,750 - 31,791 | U | Jaffré, 1980; new data |
| *Phyllanthus torrentium var induratus (P induratus)* | 8 | n.a.^b^ | 0 | 46 - 1,480 | U | Kersten *et al*., 1979 |
| *Phyllanthus vulcanii (all varieties)* | 2 | 1 | 0 | 435 - 2,235 | U | new data |
| SALICACEAE (flacourtiaceae) |  |  |  |  |  |  |
| *Casearia silvana (C. melistorum)* | 31 | n.a.^b^ | 0 | 8 - 1,490 | M | Jaffré *et al.*, 1979; new data |
| *Homalium austrocaledonicum* | 11 | 2 | 0 | 432 - 1,800 | U | Jaffré *et al.*, 1979 |
| *Homalium deplanchei* | 24 | 2 | 0 | 10 - 2,900 | M | Jaffré *et al.*, 1979; new data |
| *Homalium francii* | 7 | 5 | 2 | 1,500 - 14,500 | U | Jaffré *et al.*, 1979 |
| *Homalium guillainii* | 27 | 23 | 4 | 4,500 - 11,700 | U | Jaffré *et al.*, 1979 |
| *Homalium kanaliense var kanaliense* | 85 | 75 | 1 | 2,100 - 11,624 | U | Jaffré *et al.*, 1979; new data |
| *Homalium kanaliense var boulindae* | 2 | 1 | 0 | 675 - 1,500 | U | new data |
| *Homalium mathieuanum* | 3 | 1 | 0 | 16 - 1,690 | U | Jaffré *et al.*, 1979 |
| *Homalium rubrocostatum* | 2 | 1 | 0 | 476 - 1,160 | U | Jaffre *et al.*, 1979 |
| *Xylosma boulindae* | 4 | 2 | 0 | 722 - 1,930 | U | Jaffré *et al.*, 1979 |
| *Xylosma confusum* | 9 | n.a.^b^ | 0 | 70 - 1,630 | U | Jaffré *et al.*, 1979 |
| *Xylosma dothioense* | 4 | n.a.^b^ | 0 | 10 - 1,780 | M | Jaffré *et al.*, 1979 |
| *Xylosma kaalense* | 3 | n.a.^b^ | 0 | 1,430 - 1,900 | U | Jaffré *et al.*, 1979 |
| *Xylosma molestum* | 3 | n.a.^b^ | 0 | 565 - 1,140 | U | Jaffré *et al.*, 1979 |
| *Xylosma pancheri* | 14 | n.a.^b^ | 0 | 29 - 1,130 | M | Jaffré *et al.*, 1979 |
| *Xylosma peltatum (Lasiochlamys peltata)* | 1 | 1 | 0 | 1000 | U | Jaffré *et al.*, 1979 |
| *Xylosma pininsulare* | 2 | n.a.^b^ | 0 | 538 - 1,280 | U | Jaffré *et al.*, 1979 |
| *Xylosma serpentinum* | 7 | n.a.^b^ | 0 | 147 - 1,490 | U | Jaffré *et al.*, 1979 |
| *Xylosma tuberculatum* | 5 | n.a.^b^ | 0 | 615 - 1,600 | U | Jaffré *et al.*, 1979 |
| *Xylosma vincentii* | 10 | n.a.^b^ | 0 | 22 - 3,750 | M | Jaffré *et al.*, 1979 |
| VIOLACEAE |  |  |  |  |  |  |
| *Agatea longipedicellata (Agatea deplanchei)* | 20 | 3 | 0 | 135 - 2,500 | U | Jaffré, 1980; new data |
| *Agatea schlechteri (Agatea deplanchei)* | 6 | 1 | 0 | 300 - 1,400 | M | Jaffré, 1980; new data |
| *Hybanthus austrocaledonicus* | 53 | 1 | 52 | 6,900 - 25,500 | U | Jaffré, 1980; new data |
| *Hybanthus caledonicus* | 30 | 20 | 6 | 160 - 17,500 | M | Jaffré, 1980; new data |
| **Oxalidales** |  |  |  |  |  |  |
| CUNONIACEAE |  |  |  |  |  |  |
| *Codia spatulata* | 89 | 3 | 0 | 27 - 1,420 | M | new data |
| *Geissois bradfordii* | 2 | 1 | 1 | 6,500 - 12,707 | U | new data |
| *Geissois hirsuta* | 12 | 9 | 0 | 577 - 4,998 | M | Jaffré *et al.*, 1979; new data |
| *Geissois magnifica* | 4 | 3 | 0 | 788 - 3,250 | U | Jaffré *et al.*, 1979; new data |
| *Geissois lanceolata* | 16 | 14 | 2 | 2,620 - 22,900 | U | Jaffré *et al.*, 1979; new data |
| *Geissois pruinosa (all varieties)* | 40 | 31 | 9 | 3,000 - 15,106 | U | Jaffré *et al.*, 1979; new data |
| *Geissois velutina* | 2 | 1 | 0 | 571 - 1,085 | U | new data |
| *Pancheria alaternoides* | 68 | 3 | 0 | 23 - 1,600 | U | new data |
| *Pancheria engleriana* | 9 | 5 | 0 | 1,200 - 6,300 | U | Jaffré, 1980 |
| *Pancheria ferruginea* | 1 | 1 | 0 | 4,125 | U | new data |
| *Pancheria reticulata* | 4 | 4 | 0 | 2,025 - 3,250 | U | new data |
| **Myrtales** |  |  |  |  |  |  |
| MYRTACEAE |  |  |  |  |  |  |
| *Cloezia artensis var artensis* | 60 | 2 | 0 | 35 - 1,729 | M | new data |

| **Brassicales** |  |  |  |  |  |  |
| --- | --- | --- | --- | --- | --- | --- |
| CAPPARACEAE |  |  |  |  |  |  |
| *Capparis artensis* | 3 | 1 | 0 | 321 - 1,249 | M | new data |
| **Ericales** |  |  |  |  |  |  |
| SAPOTACEAE |  |  |  |  |  |  |
| *Pycnandra acuminata* | 10 | 0 | 10 | 11625 - 25,500 | U | Jaffré *et al.*, 1976; new data |
| *Pycnandra caeruleilatex* | 1^c^ | - | - | >1000 ^c^ | U | Swenson and Munzinger, 2010; new data |
| **Gentianales** |  |  |  |  |  |  |
| RUBIACEAE |  |  |  |  |  |  |
| *Gynochtodes sp (specimen Dagostini 87)* | 3 | 1 | 2 | 8,962 - 15,245 | U | new data |
| *Normandia neocaledonica* | 22 | 1 | 0 | 23 - 1,000 | U | new data |
| *Psychotria gabriellae (P. douarrei)* | 55 | 0 | 55 | 18,000 - 63,750 | U | Jaffré and Schmid, 1976; new data |
| **Asterales** |  |  |  |  |  |  |
| ARGOPHYLLCEAE |  |  |  |  |  |  |
| *Argophyllum ellipticum (A laxum + A. grunowi)* | 10 | 5 | 0 | 375 - 1,900 | U | Jaffré, 1977; 1980 |
| **Unplaced to order** |  |  |  |  |  |  |
| ONCOTHECACEAE |  |  |  |  |  |  |
| *Oncotheca balansae* | 4 | 4 | 0 | 1,000 - 2,500 | U | Jaffré, 1980 |

^a^ : U, taxa only found on ultramafic soils; M, taxa found on ultramafic and non-ultramafic soils.

^b^: Data were given as mean value, individual data are not available

^c^: Nickel concentration was estimated by dimethylglyoxime assay.
